# Enhanced Sensitivity of Cell Identification in Complex Environments Using Chirally Inverted L‐DNA‐Based Logic Devices

**DOI:** 10.1002/advs.202410642

**Published:** 2024-10-14

**Authors:** Zixi Lai, Di Jin, Yuan Tian, Xiaoxing Chen, Da Han, Haige Chen, Junyan Wang, Yang Yang

**Affiliations:** ^1^ Shanghai Pulmonary Hospital School of Medicine Tongji University Shanghai 200092 China; ^2^ Institute of Molecular Medicine (IMM) and Department of Urology Renji Hospital School of Medicine Shanghai Jiao Tong University Shanghai 200127 China; ^3^ Hangzhou Institute of Medicine (HIM) Chinese Academy of Sciences Hangzhou Zhejiang 310022 China; ^4^ Central Laboratory Shanghai Pulmonary Hospital School of Medicine Tongji University Shanghai 200433 China; ^5^ School of Materials Science and Engineering Tongji University Shanghai 201804 China

**Keywords:** cell identification, complex biological environments, highly biostable L‐DNA, logic devices, strict hybridization properties

## Abstract

Accurate identification and isolation of target cells are crucial for precision diagnosis and treatment. DNA aptamer‐based logic devices provide a distinct advantage in this context, as they can logically analyze multiple cell surface markers with high efficiency. However, the susceptibility of natural DNA (D‐DNA) to degradation can compromise the sensitivity and specificity of these devices, potentially leading to false‐positive and false‐negative results, particularly in complex biological environments. To address this issue, dual‐ and triple‐aptamer‐based cell‐surface logic devices are designed and developed using mirror‐image L‐DNA, a chiral molecule of D‐DNA with high biostability. These devices allow for simultaneous analysis of multiple cell surface proteins, achieving greater specificity in cell identification and isolation than D‐DNA‐based logic devices. The L‐DNA probes realized 98.7% and 70.5% sensitivities in FBS buffer with dual‐ and triple‐aptamer‐based logic devices for target cell identification, while D‐DNA probes only showed 27.9% and 0.1%. It is believed that the high stability of L‐DNA and the high efficiency of the devices for labeling cell subpopulations will have broad applications in the life sciences, biomedical engineering, and personalized medicine.

## Graphical abstract

Herein, L‐DNA is utilized as building blocks for constructing logic devices and hybridization chain reactions to identify and isolate cell subpopulations with high specificity and efficiency in complex environments, which demonstrated significant superiority in both PBS and FBS compared to D‐DNA probes, attributed to the high biostability and strict hybridization properties of L‐DNA.

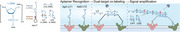

## 1 Introduction

The precise identification of cancer cells is crucial for accurate cancer diagnosis and personalized medicine. The use of cell surface proteins as molecular markers for target cell isolation is advantageous due to their accessibility and scalability.^[^ ^1^ ^]^ However, identifying small differences between similar cell subtypes poses a challenge for precisely identifying and promptly isolating target cells with a single cell surface protein.^[^ ^2a,b^ ^]^ Recent advances in DNA logic devices offer a highly effective means for achieving one‐step identification of target cells with a high degree of efficiency. These intelligent devices analyze multiple cell surface proteins simultaneously and logically, resulting in precise labeling followed by rapid isolation of cell subpopulations without the need for multiple rounds. For instance, methods including multiple‐aptamer‐based DNA logic devices via associative toehold activation,^[^ ^3^ ^]^ multiple‐aptamer‐mediated proximity ligation,^[^ ^4^ ^]^ fluidically confined CRISPR‐based DNA reporters,^[^ ^5^ ^]^ and localized DNA‐based biomolecular reaction networks^[^ ^6^ ^]^ have been developed for cell identification with high specificity. However, the inherent susceptibility of naturally occurring D‐DNA to degradation in both in vitro and in vivo settings significantly limits its practical utility as a tool for diagnostics and therapeutics. Previous studies utilized HCR‐based logic devices in D‐PBS supplemented with glucose and magnesium or conducted RCA reactions on cell membranes in Phi29 DNA polymerase reaction buffer containing 50 mM Tris‐HCl, 10 mM MgCl_2_, 10 mM (NH_4_)_2_SO_4_, and 4 mM DTT.^[^ ^3^, ^4^ ^]^ While these conditions are adequate for constructing DNA logic devices on living cell membranes for diagnostic purposes, cells labeled with DNA probes under these conditions typically take several hours and become less viable, rendering them unsuitable for further therapeutic applications.^[^ ^7^ ^]^ Additionally, the nonspecific binding of D‐DNA devices to interferent nucleic acids and proteins can be another factor that affects the sensitivity, specificity, and accuracy of D‐DNA‐based logic devices for cell identification.^[^ ^8^ ^]^ Therefore, successful DNA‐based logic gate computation on living cell membranes thus depends on cell‐friendly conditions, usually involving fetal bovine serum (FBS), where DNA‐labeled cells can be propagated further. To the best of our knowledge, there are a few DNA logic‐gate‐based methods that can accurately identify and isolate target cells in a serum‐containing environment or biological fluid.

Our goal was to address this issue by employing chirally inverted mirror‐image oligonucleotides, specifically L‐DNA and L‐RNA.^[^ ^8^, ^9^ ^]^ These enantiomers of natural nucleic acids share identical physical characteristics with D‐DNA, including solubility and base pairing capabilities. However, they distinguish themselves by forming a left‐handed double helix upon hybridization with a matching L‐DNA sequence. The key advantage of L‐DNA and L‐RNA is their resistance to nucleases, which confers an exceptional level of stability. This attribute renders them highly suitable for use in the fields of diagnostics and therapeutics.^[^ ^7^, ^10^ ^]^ Because of these properties, L‐DNA and L‐RNA have been utilized in drug assembly,^[^ ^11^ ^]^ data storage,^[^ ^12^ ^]^ aptamer selection,^[^ ^13^ ^]^ and intracellular enzyme activity analysis.^[^ ^14^ ^]^ Moreover, given the antinuclease, nontoxic, and low immunogenic nature of mirror‐image oligonucleotides, L‐DNA and L‐RNA probes have been extensively developed for therapeutic purposes.^[^ ^15^ ^]^

In this study, we employed D‐DNA aptamer‐L‐DNA scaffold hybrids as building blocks for logic devices for identifying and isolating cell subpopulations. The high affinity and specificity of D‐DNA aptamers for their targets, as well as their resistance to nuclease degradation upon target binding, make them suitable for the recognition of cell membrane proteins.^[^ ^16a–e^ ^]^ The excellent biostability of the L‐DNA scaffold, as well as its low binding affinity for environmental nucleic acids and proteins, helped us develop highly effective signal amplification methods for sensitive cell labeling, thereby ensuring the excellent accuracy of the devices for cell identification and isolation in complex environments.^[^ ^8^ ^]^ Using the L‐DNA logic device, we achieved simultaneous analysis of two and three membrane proteins, resulting in rapid and accurate cell identification in a single step. The sensitivities of the L‐DNA devices were 98.7% and 70.5% with dual‐aptamer‐ and triple‐aptamer‐based logic gates, respectively, while those of the control D‐DNA devices were 27.9% and 0.1%, respectively. We also demonstrated high diagnostic accuracy with clinical samples via an L‐DNA device via target cell identification in real blood samples. We believe that the enhanced stability of L‐DNA offers a significant advantage over traditional D‐DNA, potentially transforming the landscape of diagnostics and therapeutics by providing a robust platform for the development of advanced cell identification and isolation applications.

## 2 Results and Discussion

### 2.1 Design and Mechanism of the L‐DNA‐Based Logic Device for Cell Identification

**Figure 1:**
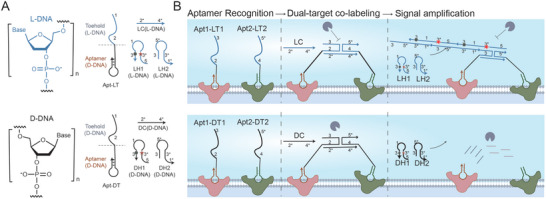
Design and mechanism of the L‐DNA‐based logic device for cell identification.

Our aim was to employ L‐DNA probes to analyze the unique expression patterns of multiple cell surface proteins to identify cell subtypes in complex environments. We have engineered an L‐DNA probe, designated as Apt‐LT, which integrates a D‐DNA aptamer for the recognition of cell surface proteins (Apt) and a single‐stranded L‐DNA segment that serves as an initiation point for the hybridization chain reaction (HCR) for signal amplification (**Figure** **1**). When the cell membrane presents the target proteins, the Apt‐LT probes engage with them via the specific interaction of the aptamers, thereby exposing distinct regions of the associative toehold sequence (Figure 1). We utilized a single‐stranded L‐DNA connector sequence (LC) to link various Apt‐LT probes through hybridization, creating an associative toehold sequence that triggers the subsequent HCR (Figure 1). To amplify the signal and achieve a superior signal‐to‐noise ratio, we synthesized fluorophore‐modified hairpins for the HCR (LH1 and LH2) using L‐DNA, resulting in a highly sensitive L‐HCR system.

It is worth noting that the absence of effective screening methods has traditionally limited the application of L‐DNA aptamers targeting cell surface proteins.^[^ ^17^ ^]^ However, we have observed that target binding can substantially increase the degradation resistance of D‐DNA aptamers by providing steric hindrance, which serves to protect the aptamers from nuclease degradation.^[^ ^18a,b^ ^]^ This innovative approach enhances the practicality and reliability of L‐DNA probes in the context of cell surface protein recognition and subsequent diagnostic applications.

### 2.2 Characterization of the L‐DNA Device for Target Cell Labeling Via a Dual‐Aptamer‐Based Logic Gate

**Figure 2:**
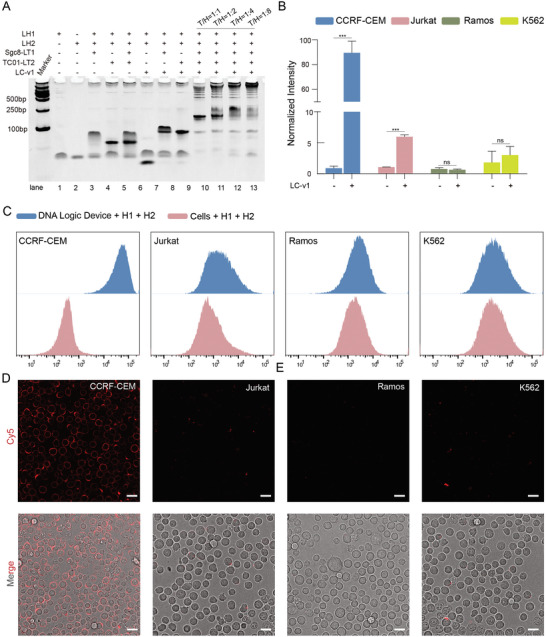
Validation of L‐DNA device for target cell labeling via a dual‐aptamer‐based logic gate. A) Native‐PAGE analysis of the dual‐aptamer‐based associative activation of HCR with L‐DNA sequences. T/H indicates the concentration ratio of toehold to HCR hairpins. B) Representative flow cytometry results of labeling signals on four different cell types using a dual‐aptamer‐based logic device with L‐DNA probes. C) Statistical analysis of flow cytometry signals for four different cell types using a dual‐aptamer‐based logic device with L‐DNA probes. p values were determined by two‐sample equal variance two‐tailed *t*‐tests (^***^ *p* < 0.001, ^**^ *p* < 0.01, ^*^ *p* < 0.05, ns = not significant). D) Confocal microscopy images of four different cell types with a dual‐aptamer‐based AND logic device using L‐DNA probes. The scale bar indicates 10 µm.

To determine the feasibility of the L‐DNA‐based logic device for cell indentation, two aptamers were utilized as the most basic models: Sgc8,^[^ ^19a,b^ ^]^ which specifically binds to the membrane receptor tyrosine‐protein kinase‐like 7 (PTK7), and TC01,^[^ ^20^ ^]^ which targets unknown overexpressed blood cancer cell surface markers. We synthesized and purified L‐DNA probes (Sgc8‐LT1‐v1, TC01‐LT2‐v1, LC‐v1, LH1‐v1, and LH2‐v1). Mass spectrometry (MS) and denaturing‐PAGE confirmed the successful synthesis and high purity of the probes (Figures S1–S6, Supporting Information). The gel electrophoresis results confirmed the assembly of the associative toehold structure and the activation of the L‐HCR in solution (**Figure** **2**A). Only the groups containing Sgc8‐LT1‐v1, TC01‐LT2‐v1, LC‐v1, LH1‐v1, and LH2‐v1 displayed obvious L‐HCR products, while the other groups lacking any of these sequences did not exhibit cascade amplification (Figure 2A). In addition, we observed more products as the concentration ratio of the trigger to HCR hairpins increased (Figure 2A). Since D‐DNA and L‐DNA share the same base‐pairing property, we also confirmed the associative activation of HCR with D‐DNA probes (Sgc8‐DT1‐v1, TC01‐DT2‐v1, DC1‐v1, DH1‐v1, and DH2‐v1). Native‐PAGE analysis revealed similar results as the D‐DNA probes (Figure S7, Supporting Information). These findings demonstrate the utility of L‐DNA sequences for the associative activation of HCR amplification for the first time.

The next phase of our research involves evaluating the performance of the L‐DNA logic device on living cell membranes. We used human acute lymphoblastic leukemia (CEM) cells, which have a dual‐positive recognition pattern for Sgc8 and TC01, as a positive cell line.^[^ ^3^ ^]^ Comparatively, the control cell lines with single‐positive or dual‐negative recognition patterns for Sgc8 and TC01 (such as human Burkitt's lymphoma cells, Ramos; human acute T leukemia cells, Jurkat; and human erythromyeloblastoid leukemia cells, K562^[^ ^3^ ^]^) exhibited minimal fluorescence enhancement, with a signal gain lower than 6 (Figure 2B,C). However, CEM cells displayed more significant fluorescence enhancement, with a signal gain of 90 (Figure 2B,C). The final signals were normalized based on the fluorescence intensity of the cells incubated with LH1‐v1 and LH2‐v1. As a result, the positive‐to‐negative ratio was ≈15. Confocal microscopy images also revealed a clear Cy5 signal on the CEM cell membrane, confirming the successful design and operation of L‐DNA AND logic devices on living cell membranes via associative‐activated HCR (Figure 2D). To be noted, that the off‐rate of traditional aptamers from target proteins is high. We connected two separate aptamers on the living cell membrane with the connecter sequence, resulting in a bivalent aptamer. Obviously, the bivalent aptamer has a slower off‐rate than each of the monovalent aptamers does. The nonspecific HCR products generated by one of the split‐triggers on the single‐aptamer‐binding negative cell would dissociate due to the high off‐rate. However, the slow off‐rate of the bivalent aptamer maintains high signal intensity. As a result, the high off‐rate of the monovalent aptamer and the slow off‐rate of the bivalent aptamer further increased the positive‐to‐negative ratio.

We also tested the D‐DNA logic device on living cell membranes to further verify the design of the logic device. By normalizing with the fluorescence intensity of the cells incubated with DH1‐v1 and DH2‐v1, CEM cells displayed more significant fluorescence enhancement, with a signal gain of ≈130, compared with the control cell lines exhibiting low fluorescence enhancement, with a signal gain of ≈20, resulting in a positive‐to‐negative ratio of 6.6 (Figure S8, Supporting Information). To be noted, the fluorescence enhancement of the L‐DNA probes was lower than that of the D‐DNA probes, the positive‐to‐negative ratio was much higher, attributing to the high biostability and strict hybridization properties of L‐DNA (Figure 2B; Figure S8, Supporting Information). Confocal microscopy images also confirmed the successful operation of D‐DNA AND logic devices on living cell membranes via associative‐activated HCR with a clear Cy5 signal on cell membranes (Figure S9, Supporting Information). These results indicated the potential of target cell identification with L‐DNA‐based logic devices due to the same base pairing property of L‐DNA and D‐DNA sequences.

### 2.3 Characterization of the L‐DNA Device for Target Cell Labeling Via a Triple‐Aptamer‐Based Logic Gate

**Figure 3:**
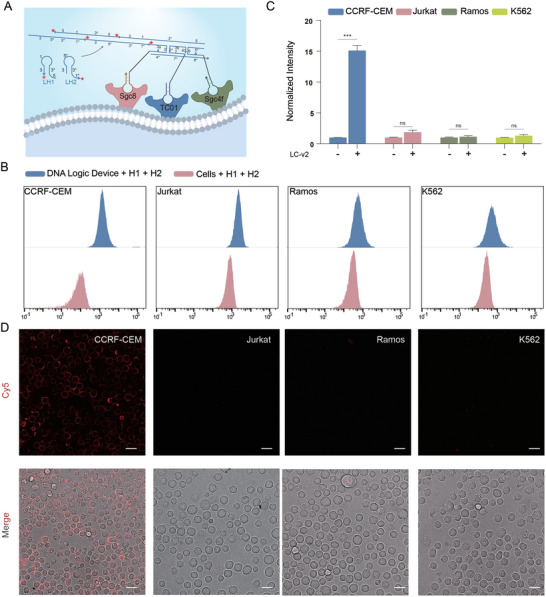
Validation of the L‐DNA device for target cell labeling via a triple‐aptamer‐based logic gate. A) Sgc8‐, TC01‐, and Sgc4f‐based AND logic devices with L‐DNA sequences. B) Representative flow cytometry results of labeling signals for four different cell types using a triple‐aptamer‐based logic device with L‐DNA probes. C) Statistical analysis of the flow cytometry signals in four different cell types using a triple‐aptamer‐based logic device with L‐DNA probes. P values were determined by two‐sample equal variance two‐tailed *t*‐tests (^***^ *p* < 0.001, ^**^ *p* < 0.01, ^*^ *p* < 0.05, ns = not significant). D) Confocal microscopy images of the four different cell types labeled with a triple‐aptamer‐based L‐DNA logic device. The scale bar indicates 10 µm.

To better assess the performance of the L‐DNA device for cell identification, we conducted a trial using a triple‐aptamer‐based AND logic gate on a living cell membrane (**Figure** **3**A). By dividing the initiator sequence of HCR into three parts, we integrated them into the aptamers Sgc8, TC01, and Sgc4f to design the L‐DNA probes Sgc8‐LT1‐v2, TC01‐LT2‐v2, Sgc4f‐LT3‐v2, and LC‐v2, respectively. The aptamer Sgc4f was employed as the third recognition element capable of binding to various leukemia cells.^[^ ^21^ ^]^ The synthesis and purification of these six probes were validated using MS and denaturing gel electrophoresis (Figures S10–S16, Supporting Information). Subsequently, we evaluated the associative activation of the HCR in solution. Native‐PAGE analysis revealed cascade amplification in the groups containing Sgc8‐LT1‐v2, TC01‐LT2‐v2, Sgc4f‐LT3‐v2, and LC‐v2 (Figure S17, Supporting Information). The absence of any of these sequences did not trigger an HCR (Figure S17, Supporting Information). We then conducted a triple‐aptamer‐based logic device test on living cell membranes using four cell lines, in which CEM demonstrated a triple‐positive recognition pattern for Sgc8, TC01, and Sgc4f.^[^ ^3^ ^]^ Flow cytometry analysis showed obvious signals in CEM cells with a signal gain of ≈15 (Figure 3B,C). This decrease in the signal gain compared with that of the dual‐aptamer‐based logic gate could be attributed to the challenges associated with simultaneously targeting the proteins of interest via three aptamers. The other cell lines that did not bind to the three aptamers simultaneously exhibited weak signals, with a signal gain of ≈2, resulting in a positive‐to‐negative ratio of 7.5 (Figure 3B,C). In addition, the absence of Sgc8‐LT1‐v2, TC01‐LT2‐v2, or Sgc4f‐LT3‐v2 produced negative signals in the CEM (Figure S18, Supporting Information). The confocal microscopy images also show significant labeling of CEM cell membranes (Figure 3D). These results suggest the potential of the L‐DNA device for cell identification using a triple‐aptamer‐based logic gate. We also used D‐DNA probes to verify the sequence design and obtained results similar to those of the L‐DNA probes, despite the higher signal gain (≈30 for CEM cells, and 10 for negative cells) and lower positive‐to‐negative ratio (3) (Figures S19 and S20, Supporting Information).

### 2.4 L‐DNA‐Based Logic Device for Cell Identification in Complex Environments

**Figure 4:**
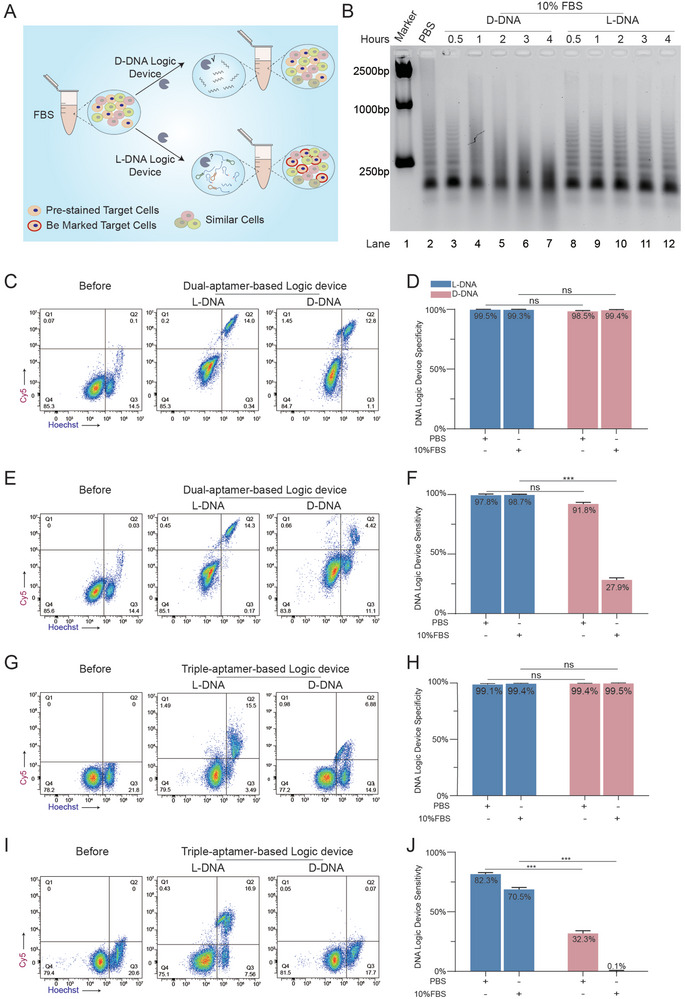
L‐DNA devices enable cell identification in complex environments. A) Schematic illustration of target cell identification in similar mixed cells. B) Native‐PAGE analysis of the HCR products constructed with D‐ or L‐DNA probes in 10% FBS. C,E) Representative flow cytometry analysis of mixed cells labeled with dual‐aptamer‐based D‐ or L‐DNA logic devices. The target cells (CEMs) were prestained with Hoechst 33 342. In situ, cell membrane HCR reactions were performed in PBS (C) or RPMI 1640 medium supplemented with 10% FBS (E). D,F) Statistical analysis of the target CEM cell identification specificities (D) and sensitivities (F) using a dual‐aptamer‐based logic device with D‐DNA and L‐DNA probes in PBS and 10% FBS. The sensitivity was calculated as the ratio of Q2/(Q2 + Q3). The specificity was calculated as the ratio of Q4/(Q1 + Q4). G,I) Representative flow cytometry analysis of mixed cells labeled with a triple‐aptamer‐based D‐ or L‐DNA logic device. The target cells (CEMs) were prestained with Hoechst 33 342. In situ, cell membrane HCR reactions were performed in PBS (G) or RPMI 1640 medium supplemented with 10% FBS (I). H,J) Statistical analysis of the target CEM cell identification specificities (H) and sensitivities (J) using a triple‐aptamer‐based logic device with D‐DNA and L‐DNA sequences in PBS and 10% FBS. The sensitivity was calculated as the ratio of Q2/(Q2 + Q3). The specificity was calculated as the ratio of Q4/(Q1 + Q4): *p* values were determined via two‐sample equal variance two‐tailed *t*‐tests (^***^ *p* < 0.001, ^**^ *p* < 0.01, ^*^ *p* < 0.05, ns = not significant).

After confirming the effectiveness of the L‐DNA probes in the logical labeling of target cells, we assessed their ability to selectively identify cell subtypes in large populations of similar cells in complex environments (**Figure** **4**A). The gel image showed the efficient assembly and high stability of HCR products with L‐DNA sequences after 4 h of incubation in 10% FBS, whereas D‐DNA HCR products gradually degraded over time (Figure 4B). We then stained the target CEM cells with Hoechst 33342, a live cell fluorescent dye, and mixed the prestained CEM cells with similar cells (Ramos, Jurkat, and K562). Dual‐aptamer‐based logic devices with D‐DNA or L‐DNA sequences for cell identification were constructed in PBS and cell culture medium supplemented with 10% FBS, respectively. Flow cytometry analysis revealed similar and highly sensitive dual‐aptamer‐based logic devices with D‐DNA and L‐DNA sequences in PBS (91.8% for D‐DNA, 97.8% for L‐DNA) (Figure 4C,F). The specificities of D‐DNA‐ and L‐DNA‐based probes were also similar and high 98.5% for D‐DNA, 99.5% for L‐DNA) (Figure 4C,D). These results indicate the excellent performance of the logic device for narrowing down cell subpopulations in mixed similar cells. However, when the logic devices were operated in a cell culture medium supplemented with 10% FBS, the L‐DNA‐based logic device exhibited obvious advantages over D‐DNA (Figure 4E,F). Despite the similar specificities (99.4% for D‐DNA, 99.3% for L‐DNA), the sensitivities of the dual‐aptamer logic devices were 98.7%, and 27.9% for L‐DNA and D‐DNA probes, respectively, which indicated the importance of the biostability of probes for operation in complex environments (Figure 4D,F). We also performed target‐cell identification in mixed‐cell populations using a triple‐aptamer‐based logic device. Using this logic device, the L‐DNA probes demonstrated significant superiority in both PBS and FBS. The sensitivities of the L‐DNA device in PBS and FBS were 82.3% and 70.5%, respectively, which are greater than those of the D‐DNA devices despite the similar specificities of both devices (Figure 4G–J). Afterward, we performed L‐DNA device‐based target cell identification in real samples by mixing different concentrations of target CEM cells into the blood. We achieved high sensitivity and specificity of target cell identification even with 50 000 cells per mL (Figure S21, Supporting Information). These results suggest that the biostability and strict hybridization properties of L‐DNA sequences significantly improved the performance of complex DNA‐based logic devices in real samples, with better reliability and accuracy than D‐DNA. Therefore, L‐DNA‐based logic devices have great potential for target‐cell identification and isolation in complex environments.

## 3 Conclusion

Advancements in DNA logic devices have significantly improved the sensitivity and accuracy of target‐cell identification and isolation.^[^ ^22^ ^]^ We and other groups have developed diverse DNA‐based logic devices for autonomous and logical analysis of the unique expression patterns of multiple membrane proteins in various cell types. However, the ease of inactivation caused by degradation or nonspecific binding to nucleic acids and proteins in complex environments greatly limits their practical application in the biomedical field. Nonnatural nucleic acids, such as PNA, XNA, L‐DNA, and L‐RNA, have been widely used for designing probes. However, the synthesis of PNA or XNA is complicated, and the cost is high. In this study, we used L‐DNA to construct logical devices for identifying cell subpopulations in complex environments. As a chiral molecule of D‐DNA, L‐DNA exhibits high stability in serum and does not hybridize with complementary D‐DNA sequences in the environment, making it an ideal candidate for diagnostic and therapeutic applications. The stability of L‐DNA probes in biological samples could enable more reliable detection of biomarkers or pathogens. Additionally, L‐DNA aptamers and nanostructures could have a longer half‐life in the bloodstream because of their resistance to degradation, potentially leading to more effective drug delivery systems and targeted therapies for diseases such as cancer and autoimmune disorders, minimizing off‐target effects and increasing the therapeutic index of treatments. Although there are limitations in identifying L‐DNA aptamers that target cell surface proteins, we successfully performed AND logical analysis of two and three membrane proteins in living cells using D‐DNA aptamers and L‐DNA logic devices. These devices showed excellent performance in the identification of target cells in a mixture of multiple similar cell populations in a cell culture medium supplemented with 10% FBS. However, D‐DNA devices not only are unstable but also show poor performance in identifying target cells. With the dual‐aptamer‐based AND logic device, L‐DNA probes exhibit a sensitivity of 98.7% for target cell identification in the FBS buffer, which is much greater than that of D‐DNA. Additionally, L‐DNA probes revealed 82.3% and 70.5% sensitivity in PBS and FBS buffer, respectively, when using a triple‐aptamer‐based AND logic device compared with 32.3% and 0.1% sensitivity, respectively, for D‐DNA probes. The identification of target cells in real blood samples further demonstrated the high diagnostic accuracy of the L‐DNA device. We believe that the stability of L‐DNA and the precision and convenience of logic devices make this integrated approach involving L‐DNA‐based multi‐aptamers more advantageous than current cellular identification systems based on D‐DNA sequences. Our work presents a proof of principle for the isolation of target cells using an L‐DNA‐based logic gate under optimal conditions for cell growth to minimize damage to cells caused by previous methods, which are typically conducted in an FBS‐free buffer. In future studies, L‐DNA probes can be conjugated to antibodies that recognize cell surface markers to expand the application prospects of L‐DNA logic devices. Owing to the harmlessness of L‐DNA‐based logic devices, isolated cells can be cultured in an artificial medium for downstream applications, such as omics analysis, drug screening, and immunotherapy. For example, L‐DNA can be conjugated to antibodies to construct L‐DNA logic devices for isolating target T cells from whole blood samples for CAR‐T‐cell preparation, which is critical for preserving the viability of target T cells during cell isolation since the number of isolated T cells will further increase, and T cells will be infected with viruses for diagnostic treatment.

## 4 Experimental Section

### Reagents and Cell Lines

MgCl_2_, Tris‐Borate‐EDTA (TBE) buffer, gelred, acryl/bis 30% solution (29:1), D‐glucose, D‐PBS, and all the D‐DNA strands were purchased from Shanghai Sangong Corporation (Shanghai, China). Hoechst 33 342, tetramethylethylenediamine (TEMED), and ammonium persulfate (APS) were purchased from Merck Millipore (Darmstadt, Germany). Bovine serum albumin (BSA), sterile‐filtered water treated with diethyl pyrocarbonate (DEPC), and agarose were purchased from Shanghai Hongsheng Bioengineering Corporation (Shanghai, China). All the cell lines used were obtained from the Cell Bank/Stem Cell Bank, Chinese Academy of Sciences (Shanghai, China). CCRF‐CEM, Jurkat (Clone E6‐1), Ramos, and K562 cells were cultured in RPMI 1640 medium supplemented with 10% fetal bovine serum (HyClone, South Logan, USA) and 1% penicillin‒streptomycin (HyClone, South Logan, USA) at 37 °C with 5% CO_2_. Binding buffer was prepared with D‐PBS supplemented with 0.1 mg mL^−1^ herring sperm DNA (Thermo Fisher Scientific, USA), 1 mg mL^−1^ BSA, 4.5 g L^−1^ D‐glucose, and 5 mM MgCl_2_. Wash buffer was prepared with D‐PBS supplemented with 4.5 g L^−1^ D‐glucose and 5 mM MgCl_2_.

### Formation of Dual/Triple‐Aptamer‐Based Logic Devices in Buffers

All the logic devices were first verified by agarose gels in D‐PBS supplemented with 5 mM MgCl_2_. Both the L‐DNA and D‐DNA versions of Sgc8‐T1, TC01‐T2 (and Sgc4f‐T3 for triple aptamer‐based and logic devices) and the connector strand were first separately dissolved at a concentration of 10 nM in D‐PBS supplemented with 12.5 mM MgCl_2_, heated to 95 °C for 3 min, and cooled to 4 °C. Afterward, all these strands were mixed together at a final concentration of 1 nM for each component in D‐PBS supplemented with 12.5 mM MgCl_2_ to form an AND logic device at 4 °C for 30 min. H1 and H2 strands at various concentrations were then added to the mixture, followed by a 30 min incubation at 4 °C. The final product was analyzed by electrophoresis (10% polyacrylamide gel (PAGE) or 3% agarose gel) at 4 °C in TBE buffer followed by GelRed staining.

### Formation of a Dual‐Aptamer‐Based Logic Device in FBS‐Containing Buffer

L‐DNA‐containing aptamers (1 nM), connectors (1 nM), H1 (1 nM), and H2 (1 nM) strands were mixed in RPMI 1640 cell culture medium supplemented with 10% FBS and 5 mM MgCl_2_ for 0.5, 1, 2, 3, and 4 h for HCR. Similarly, D‐DNA versions of the aptamers, connector, H1 strands, and H2 strands were incubated for various durations in the same buffer. As a control, the abovementioned D‐DNA strands at the same concentration were incubated in D‐PBS supplemented with 5 mM MgCl_2_. The L‒DNA and D‒DNA versions of the HCR products were analyzed by electrophoresis.

### Flow Cytometry Analysis

For the L‐DNA system, L‐DNA containing Sgc8‐T1 (500 nM) and TC01‐T2 (500 nM) (and Sgc4f‐S‐T3 for the triple aptamer‐based logic device) was first incubated with 5 × 10^5^ CCRF‐CEM (or Ramos, K562, Jurkat) cells for 30 min at 4 °C in 100 µL of binding buffer. The cells were then washed twice with 500 µL of wash buffer, centrifuged at 1000 rpm for 3 min, and resuspended in 100 µL of wash buffer. L‐Connector (1 µM), H1 (1 µM), and H2 (1 µM) were added to the mixture, followed by a 90 min incubation. The D‒DNA version of the logic device was constructed as described above. After being washed twice, the cells were analyzed with a flow cytometer (Beckman Coulter Life Sciences, Indianapolis, IN, USA), and 10 000 events were counted at a time. FlowJo software (V10.0.81) was used for the data analysis. The positive‐to‐negative ratio was calculated by dividing the flow cytometry signal enhancement of the positive cell line by that of the negative cell line.

### Confocal Microscopy Imaging of Cells

CCRF‐CEM, Ramos, K562, and Jurkat cells were sequentially incubated with the L‐DNA versions of the aptamers, connector, H1 strands, and H2 strands at 4 °C in 1.5 mL Eppendorf tubes as described in the “Flow cytometry analysis” section. Similarly, the cells were incubated with the D‒DNA version of the logic device components. The cells were then cultured in polylysine‐treated confocal dishes (Nest, China) for 30 min at 25 °C, followed by three washes with wash buffer. The cells were finally imaged with a laser scanning microscope (TCS SP8, Leica, Germany).

### Target Cell Identification from the Cell Mixture

To investigate whether the logic gate device allows the identification of target cells from the cell mixture, CCRF‐CEM cells were first stained with Hoechst at 37 °C for 15 min. The pre‐stained cells were then washed 5 times with 1 mL of D‐PBS and mixed with Ramos and K562 cells. This cell mixture was further sequentially incubated with the L‐DNA or D‐DNA versions of the logic‐gate components in the binding buffer as described above. To evaluate the FBS stability of the logic device, the cell mixture was incubated with binding buffer supplemented with 10% FBS, followed by sequential labeling of the L‐DNA or D‐DNA versions of the logic‐gate components. The cell mixture was analyzed via flow cytometry.

### DNA Synthesis Procedure

To synthesize the D‐DNA/L‐DNA chimera, 1000 Å CPG powder (20 g) was weighed and placed on a DNA synthesis column, with the two ends of the synthesis column fixed with plug plates. The concentration of the phosphoramidite monomer solution should be 0.1 m. The D‐DNA phosphoramidite monomer reagent was then added at the designated position according to the manufacturer's instructions for the nucleic acid synthesizer. At this point, the synthesis should be paused at the last D‐DNA base. The original base vials were then removed, followed by cleaning of the pipeline. The L‐DNA phosphoramidite monomer reagent was then added, and the synthesis procedure continued until the end of the procedure.

The powder present in the synthesis column was poured into a 5 mL centrifuge tube, and 1 mL of AMA solution (ammonium hydroxide: 40% methylamine = 1:1) was added. The mixture was then incubated at 65 °C for 30 min. Afterward, the supernatant was transferred to a new centrifuge tube. Another 0.1 mL of 3 M NaCl and 2.5 mL of cold ethanol were then added to the supernatant, followed by full shaking. The samples were then transferred to a refrigerator at −20 °C for 1 h. The DNA products were precipitated by centrifugation at 12 000 rpm for 5 min. The samples were redissolved in 400 µL of 0.1 m TEAA (triethylamine acetate, Thermo Fisher Co.). The purification process was conducted via high‐performance liquid chromatography (HPLC) (Model 1620, Agilent Co.). The target peak solution was collected for freeze‐vacuum drying. The dried DNA powder was then dissolved in 200 µL of 80% acetic acid and left at room temperature for 20 min. A volume of 500 µL of cold ethanol and 20 µL of 3 m NaCl were added to the centrifuge tube, which was then vortexed and shaken. The tube was subsequently transferred to a refrigerator where it was frozen at −20 °C for 60 min. After removal of the tube, the DNA products were precipitated via centrifugation at 12 000 rpm for 5 min. The DNA mixture was redissolved in H_2_O and desalted via a desalting column.

## Conflict of Interest

The authors declare no conflict of interest.

## Supporting information



Supporting Information

## Data Availability

Research data are not shared.
