# Supplementary material for: Enhanced Sensitivity of Cell Identification in Complex Environments Using Chirally Inverted L‐DNA‐Based Logic Devices
Source: Adv Sci (Weinh). 2024 Oct 14;11(45):2410642. doi: 10.1002/advs.202410642 (PMC11615743; doi:10.1002/advs.202410642)
Supplement: Supplementary file 1 — Supporting Information [file ADVS-11-2410642-s001.docx]

Supporting Information

**Enhanced Sensitivity of Cell Identification in Complex Environments Using Chirally Inverted L-DNA-based Logic Devices**

*Zixi Lai^1,2^‡, Di Jin^2^‡, Yuan Tian^2^‡, Xiaoxing Chen^2^, Da Han^2,3^, Haige Chen^2^*, Junyan Wang^3^*, Yang Yang^1^**

(# Zixi Lai, Di Jin and Yuan Tian contributed equally to this work.)

**Table S1. All the DNA sequences used in this study**

| Name | Sequences |
| --- | --- |
| DH1-v1 | ATGAAGGACGATGTATGCT/Cy5dT/AGGGTCGACTTCCATAGACCC/BHQ3dT/AAGCATACAT |
| DH2-v1 | GACCCTAAGCATACATCGTCCTTCATATGTATGCTTAGGGTCTATGGAAGTC |
| LH1-v1 | ATGAAGGACGATGTATGCT/Cy5dT/AGGGTCGACTTCCATAGACCC/BHQ3dT/AAGCATACAT |
| LH2-v1 | GACCCTAAGCATACATCGTCCTTCATATGTATGCTTAGGGTCTATGGAAGTC |
| Sgc8-DT1-v1 | **ATCTAACTGCTGCGCCGCCGGGAAAATACTGTACGGTTAGA**tttttttttttttttCGACATCTAACCTGGCGTCCTTCAT |
| TC01-DT2-v1 | GACCCTAAGCATACATGCTCACTGACGCTAGGttttttttttttttt**ACCAAACACAGATGCAACCTGACTTCTAACGTCATTTGGTG** |
| DC-v1 | CTTACAACACCTAGCGTCAGTGAGCCCAGGTTAGATGTCG |
| Sgc8-LT1-v1 | **ATCTAACTGCTGCGCCGCCGGGAAAATACTGTACGGTTAGA**tttttttttttttttCGACATCTAACCTGGCGTCCTTCAT |
| TC01-LT2-v1 | GACCCTAAGCATACATGCTCACTGACGCTAGGttttttttttttttt**ACCAAACACAGATGCAACCTGACTTCTAACGTCATTTGGTG** |
| LC-v1 | CTTACAACACCTAGCGTCAGTGAGCCCAGGTTAGATGTCG |
| Sgc8-DT1-v2 | **ATCTAACTGCTGCGCCGCCGGGAAAATACTGTACGGTTAGA**tttttttttttttttCGGTGCAACGATCACTGGTCCCTTCAT |
| TC01-DT2-v2 | **ACCAAACACAGATGCAACCTGACTTCTAACGTCATTTGGTG**tttttttttttttttGAACGCTGAGACTGGGATTAAGCATACATCCAGTGATC |
| Sgc4f-DT3-v2 | GACCCTAATCCCAGGTACTAACGttttttttttttttt**ATCACTTATAACGAGTGCGGATGCAAACGCCAGACAGGGGGACAGGAGATAAGTGA** |
| DC-v2 | CTTACAACACGTTAGTACTCTCAGCGTTCGTTGCACCG |
| Sgc8-LT1-v2 | **ATCTAACTGCTGCGCCGCCGGGAAAATACTGTACGGTTAGA**tttttttttttttttCGGTGCAACGATCACTGGTCCCTTCAT |
| TC01-LT2-v2 | **ACCAAACACAGATGCAACCTGACTTCTAACGTCATTTGGTG**tttttttttttttttGAACGCTGAGACTGGGATTAAGCATACATCCAGTGATC |
| Sgc4f-LT3-v2 | GACCCTAATCCCAGGTACTAACGttttttttttttttt**ATCACTTATAACGAGTGCGGATGCAAACGCCAGACAGGGGGACAGGAGATAAGTGA** |
| LC-v2 | CTTACAACACGTTAGTACTCTCAGCGTTCGTTGCACCG |
| DH1-v2 | ATGAAGGACGATGTATGCTTAGGGTCGACTTCCATAGACCCTAAGCATACAT/Cy5/ |
| DH2-v2 | GACCCTAAGCATACATCGTCCTTCATATGTATGCTTAGGGTCTATGGAAGTC/Cy5/ |
| LH1-v2 | ATGAAGGACGATGTATGCTTAGGGTCGACTTCCATAGACCCTAAGCATACAT/Cy5/ |
| LH2-v2 | GACCCTAAGCATACATCGTCCTTCATATGTATGCTTAGGGTCTATGGAAGTC/Cy5/ |

The L-DNA sequences are underlined. The aptamer sequences are bold.

**Table S2. The raw material prices for L-DNA and D-DNA probe synthesis from Glen Research**

| Catalog number | For L-DNA synthesis | Size | Price |
| --- | --- | --- | --- |
| 10-2101-05 | beta-L-Pac-dA-CE Phosphoramidite | 0.5 g | $700 |
| 10-2115-05 | beta-L-Ac-dC-CE Phosphoramidite | 0.5 g | $350 |
| 10-2121-05 | beta-L-iPr-Pac-dG-CE Phosphoramidite | 0.5 g | $700 |
| 10-2130-05 | beta-L-dT-CE Phosphoramidite | 0.5 g | $350 |
| Catalog number | For D-DNA synthesis | Size | Price |
| 10-1029-10 | dmf-dG-CE Phosphoramidite | 1.0 g | $60 |
| 10-1030-10 | dT-CE Phosphoramidite | 1.0 g | $60 |
| 10-1015-10 | Ac-dC-CE Phosphoramidite | 1.0 g | $60 |
| 10-1000-10 | dA-CE Phosphoramidite | 1.0 g | $60 |


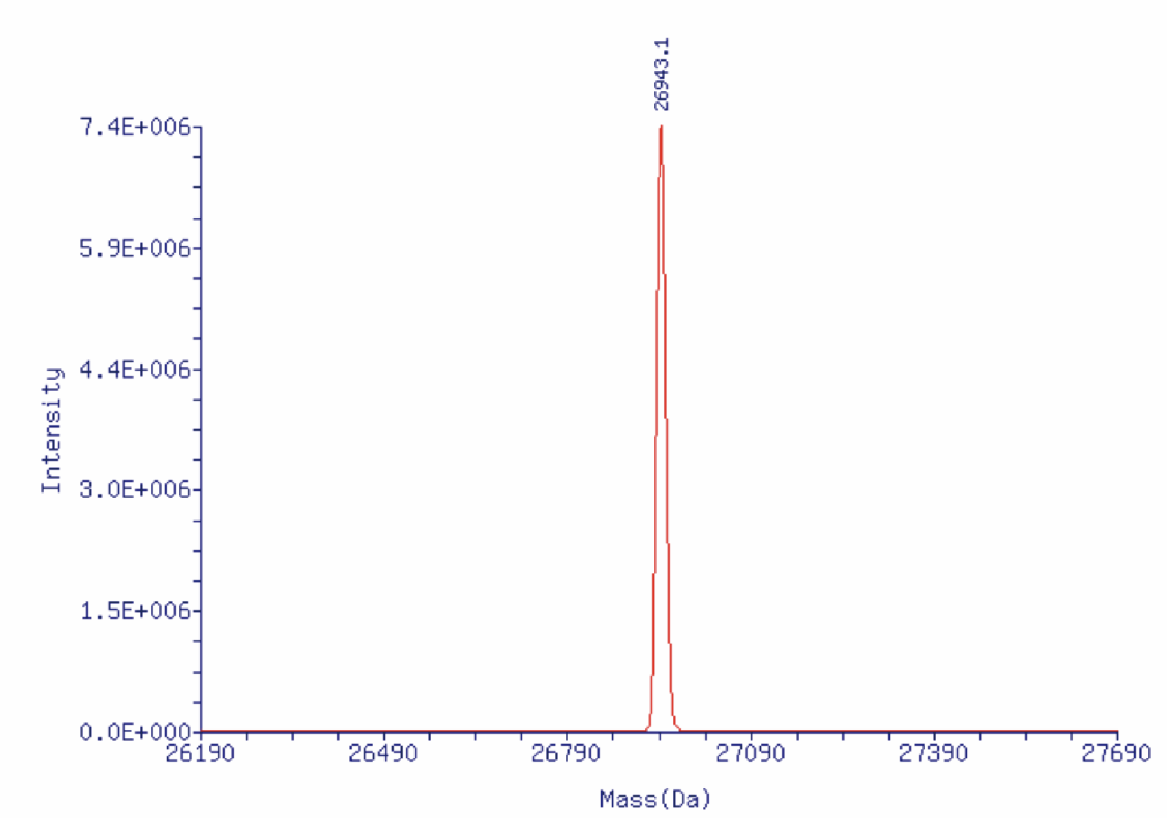


**Figure S1.** Mass spectrometry analysis of Sgc8-LT1-v1. Molecular weight is 26043.1 g/mol.


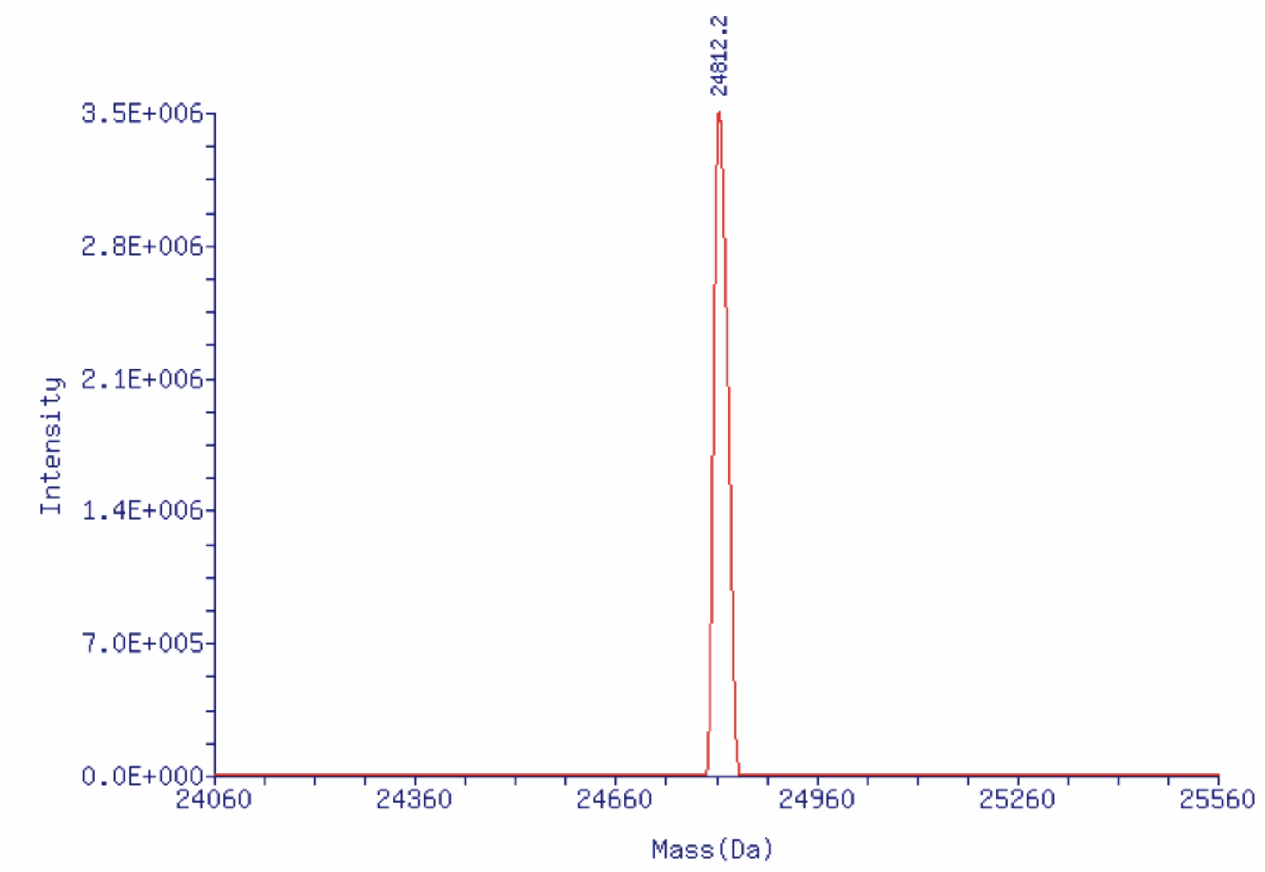


**Figure S2.** Mass spectrometry analysis of TC01-LT2-v1. Molecular weight is 24812.2 g/mol.


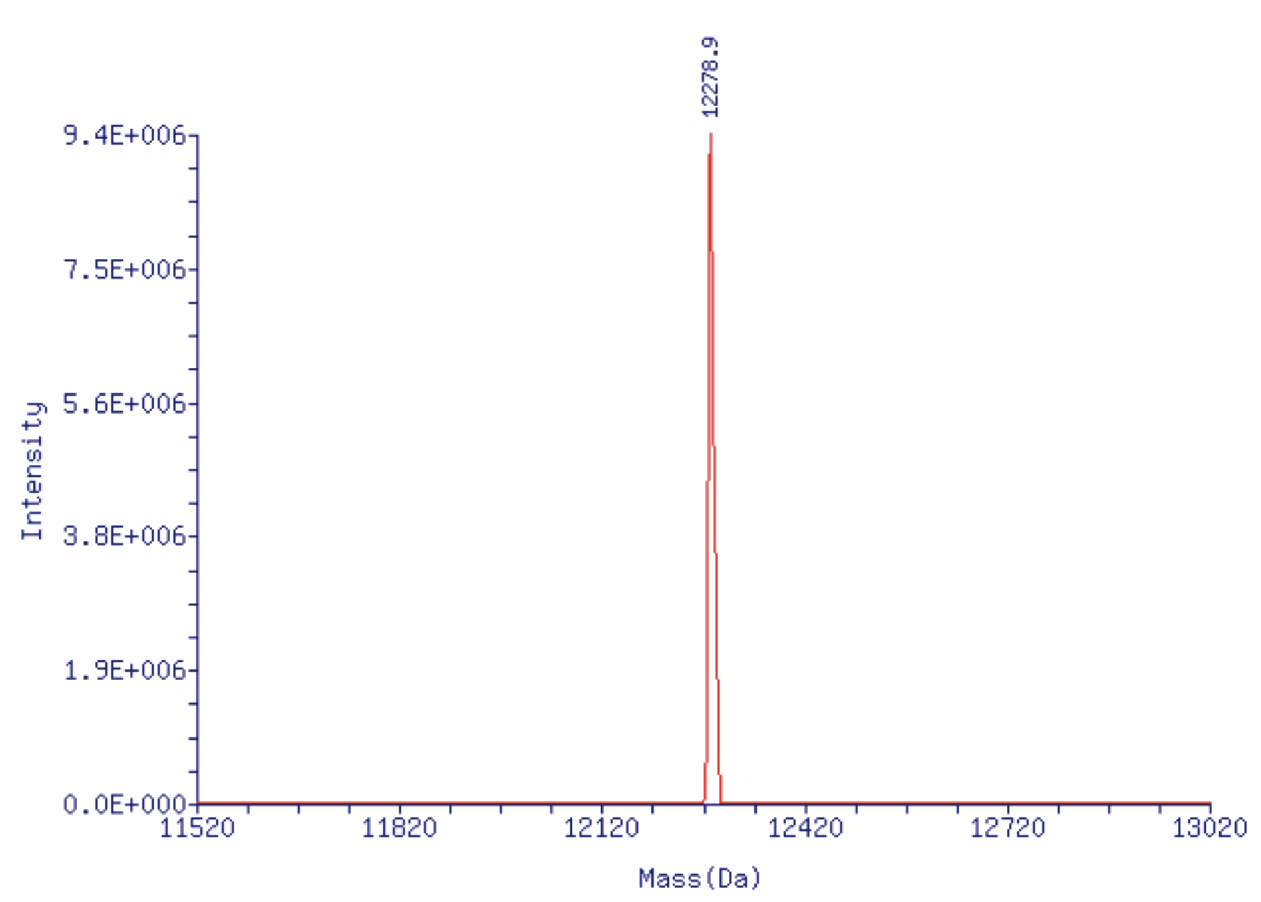


**Figure S3.** Mass spectrometry analysis of LC-v1. Molecular weight is 12278.9 g/mol.


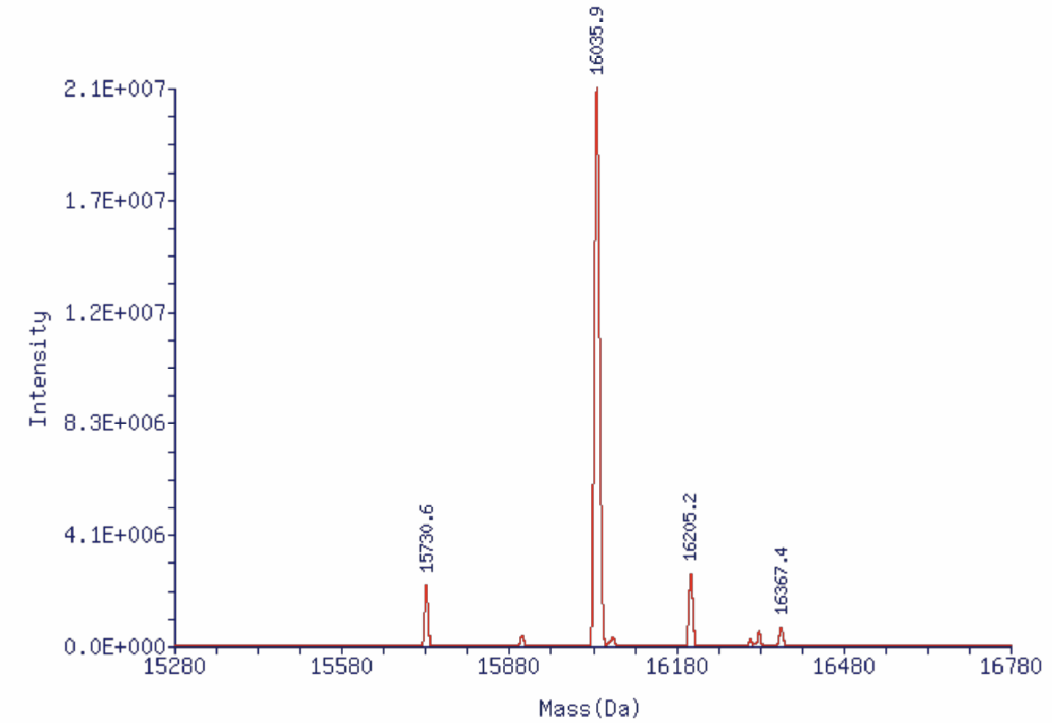


**Figure S4.** Mass spectrometry analysis of LH1-v1. Molecular weight is 16035.9 g/mol.


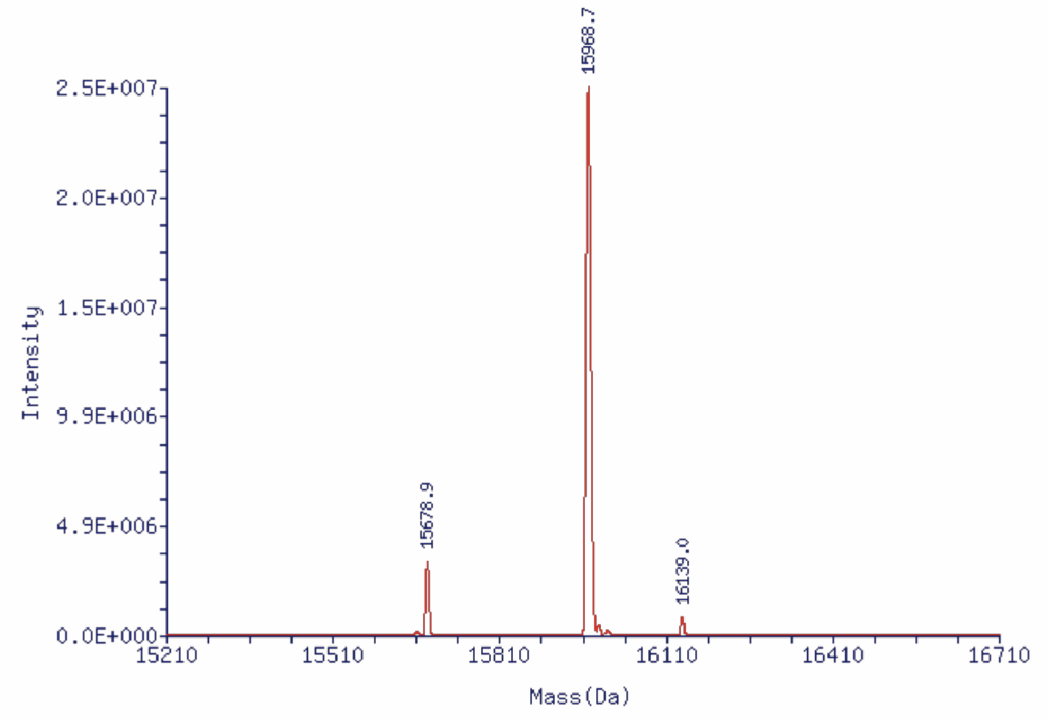


**Figure S5.** Mass spectrometry analysis of LH2-v1. Molecular weight is 15968.7 g/mol.


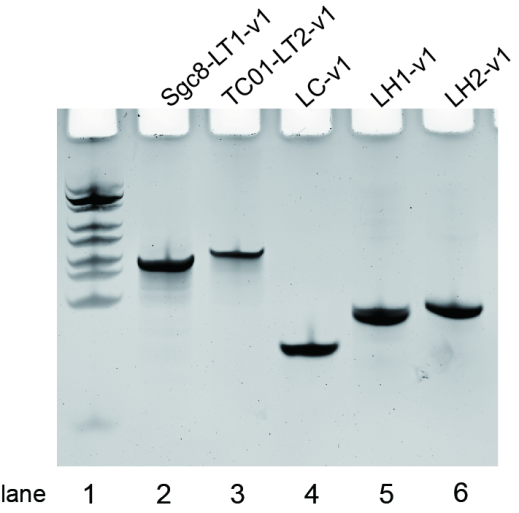


**Figure S6.** Urea PAGE analysis of L-DNA sequences of dual-aptamer-based logic device.


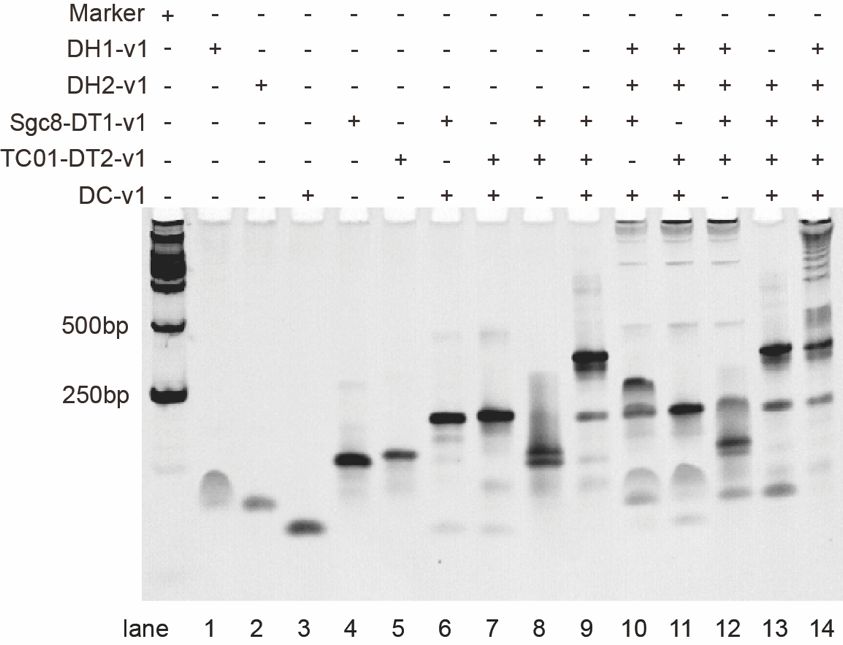


**Figure S7.** Gel electrophoresis images of D-DNA version of dual-aptamer-based HCR in solution.


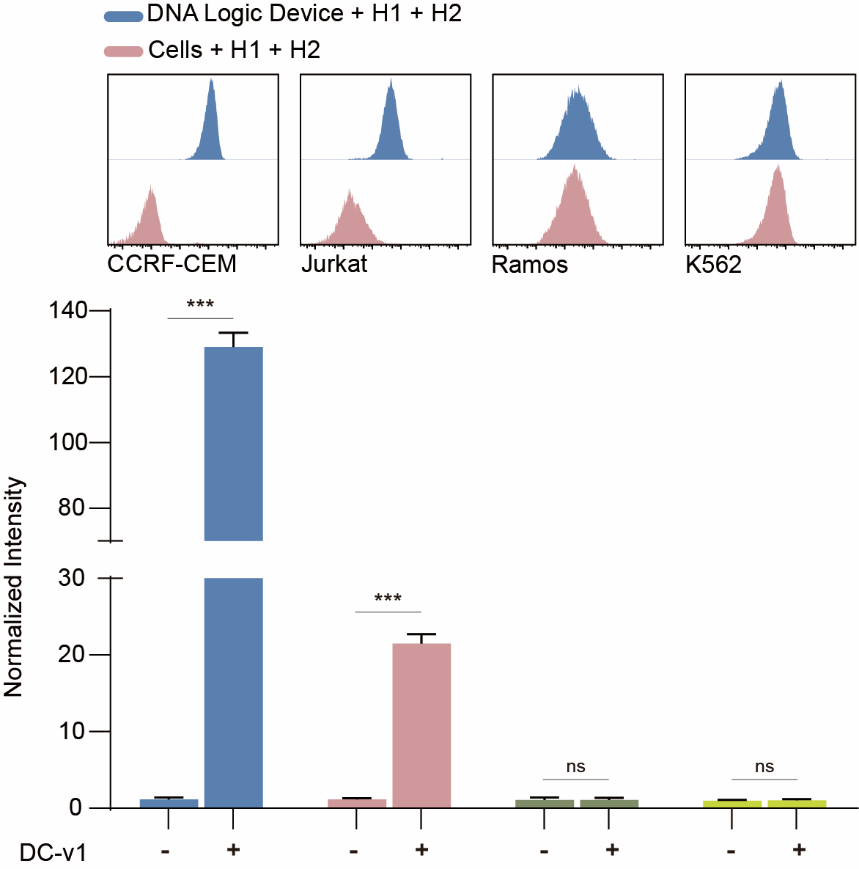


**Figure S8.** Representative and statistical analysis flow cytometry results of labeling signals on four different cell types using a dual-aptamer-based logic device with D-DNA probes. P values were determined by two-sample equal variance two-tailed t-tests (***P < 0.001, **P < 0.01, *P < 0.05, ns = not significant).


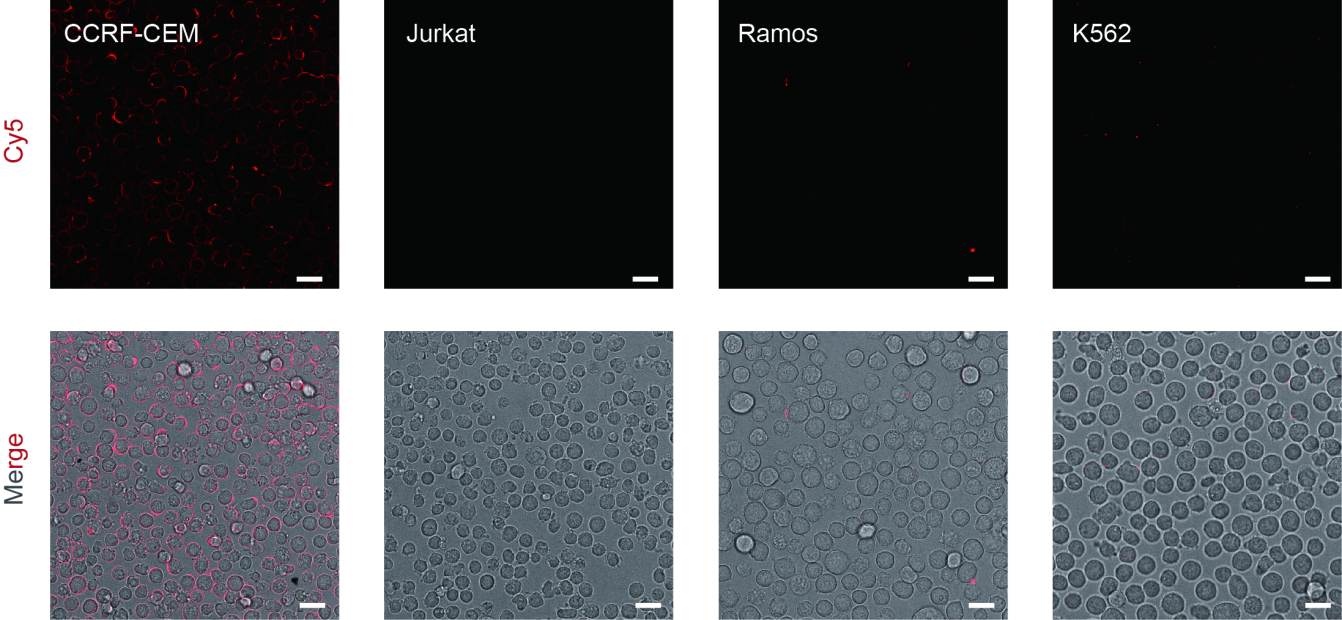


**Figure S9.** Confocal microscopy images of four different cell types with a dual-aptamer-based logic device using D-DNA probes. Scale bar indicates 10 μm.


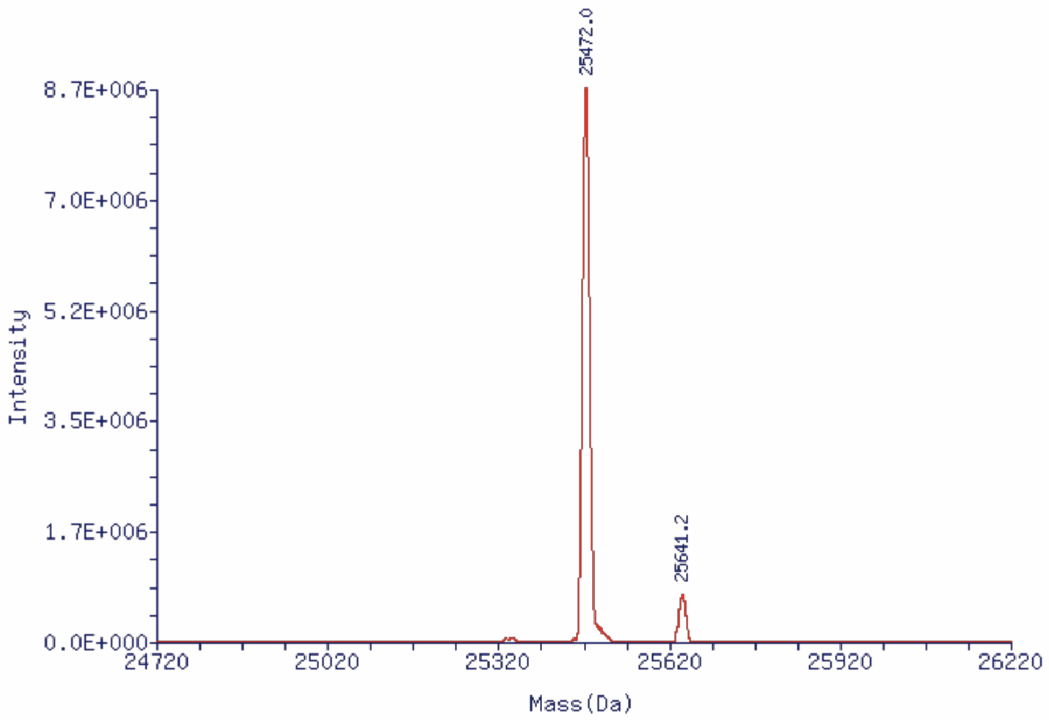


**Figure S10.** Mass spectrometry analysis of Sgc8-LT1-v2. Molecular weight is 25472.0 g/mol.


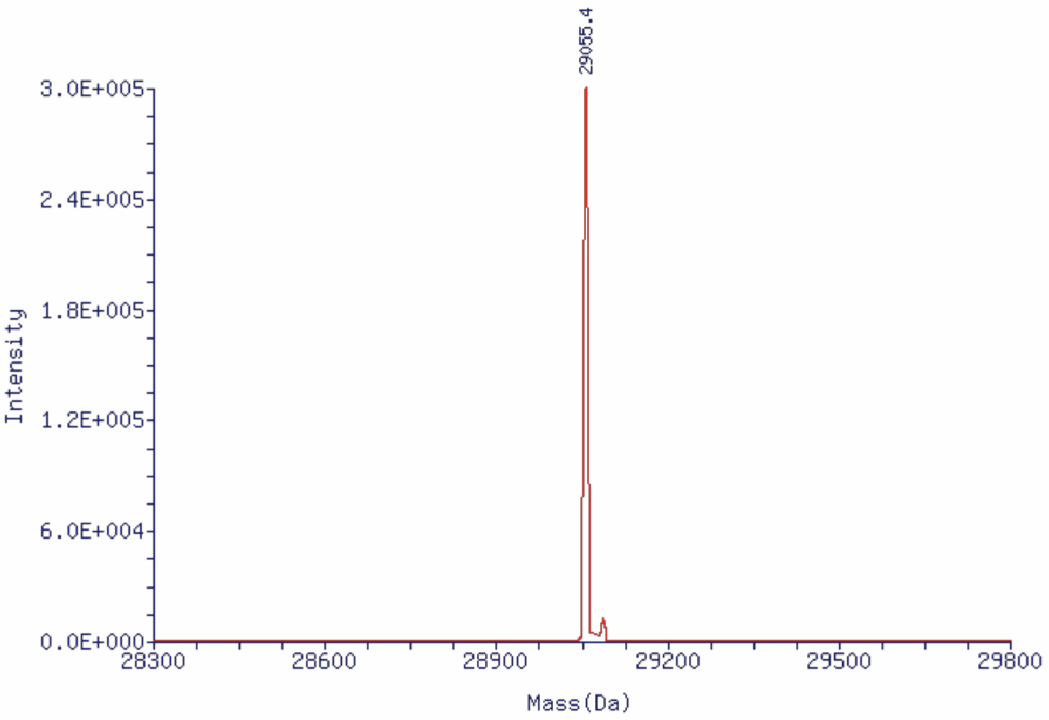


**Figure S11.** Mass spectrometry analysis of TC01-LT2-v2. Molecular weight is 29055.4 g/mol.


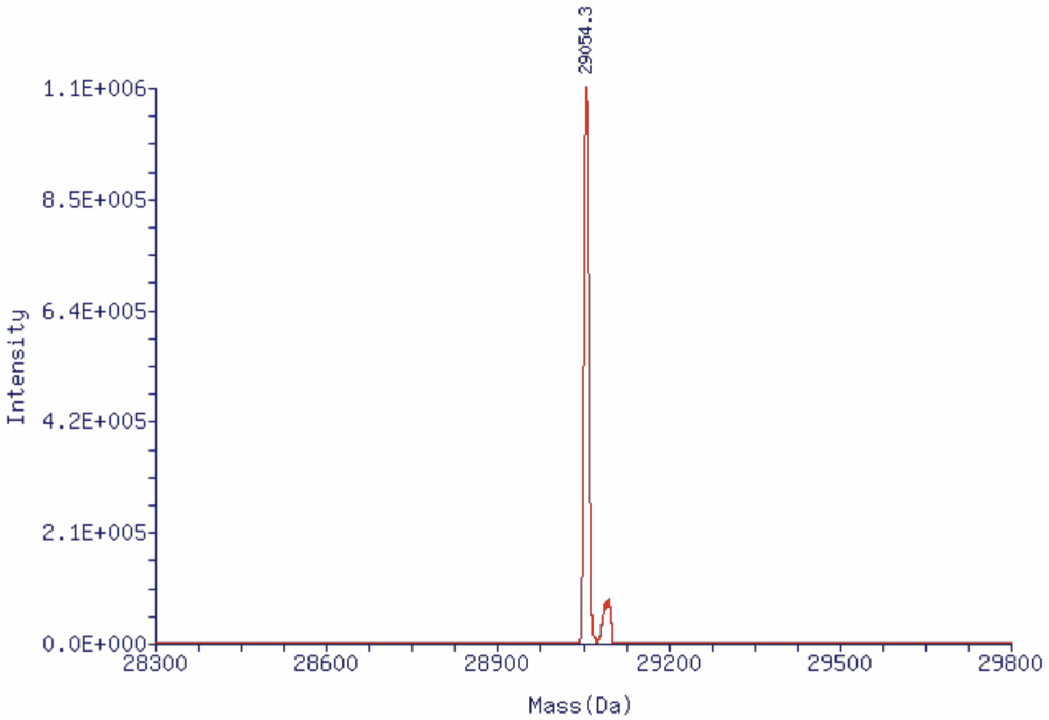


**Figure S12.** Mass spectrometry analysis of Sgc4f-LT3-v2. Molecular weight is 29054.3 g/mol.


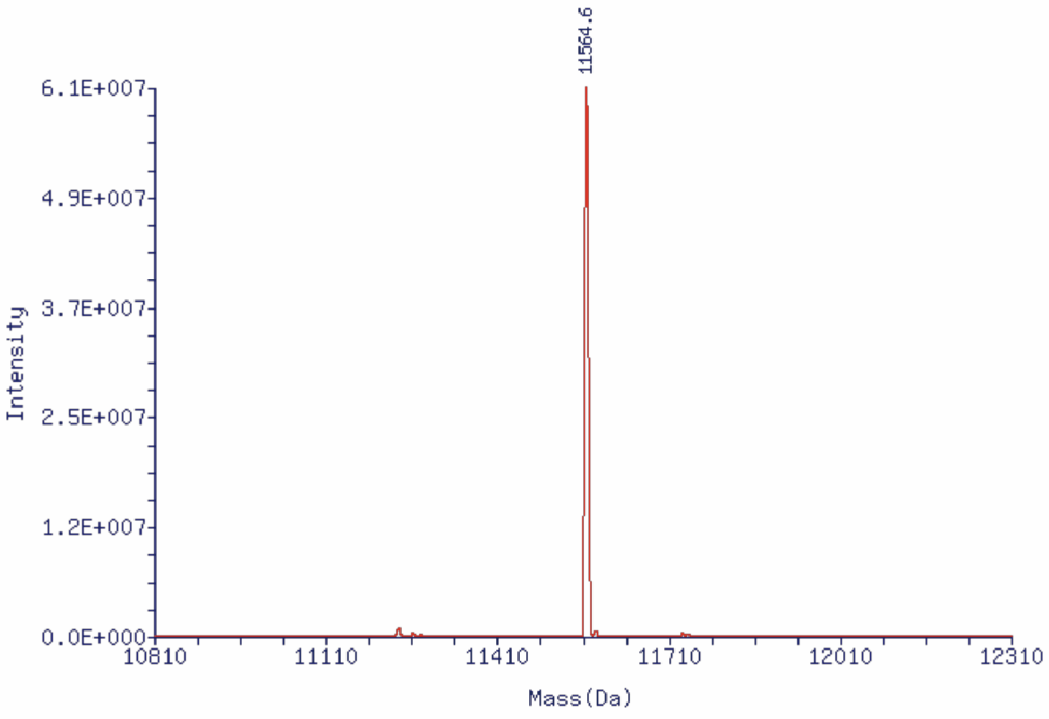


**Figure S13.** Mass spectrometry analysis of LC-v2. Molecular weight is 11564.6 g/mol.


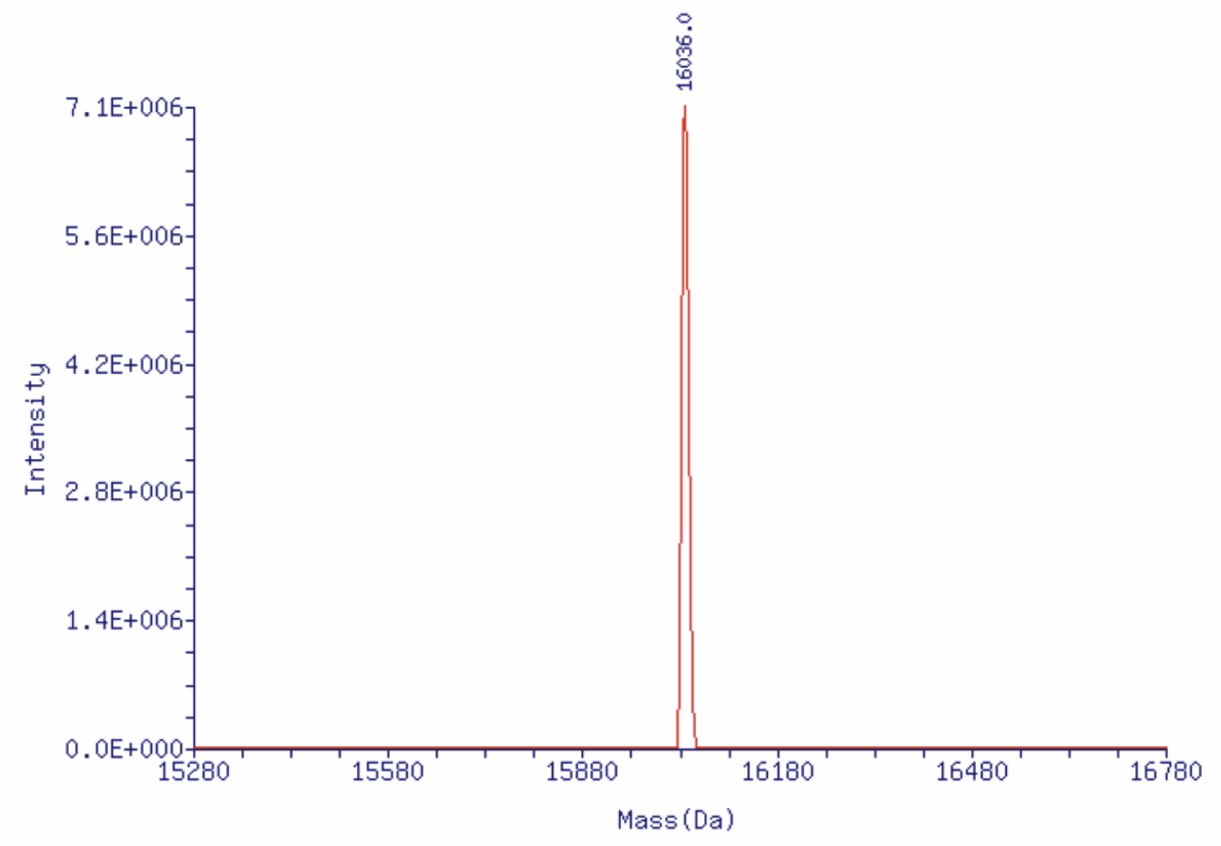


**Figure S14.** Mass spectrometry analysis of LH1-v2. Molecular weight is 16036.0 g/mol.


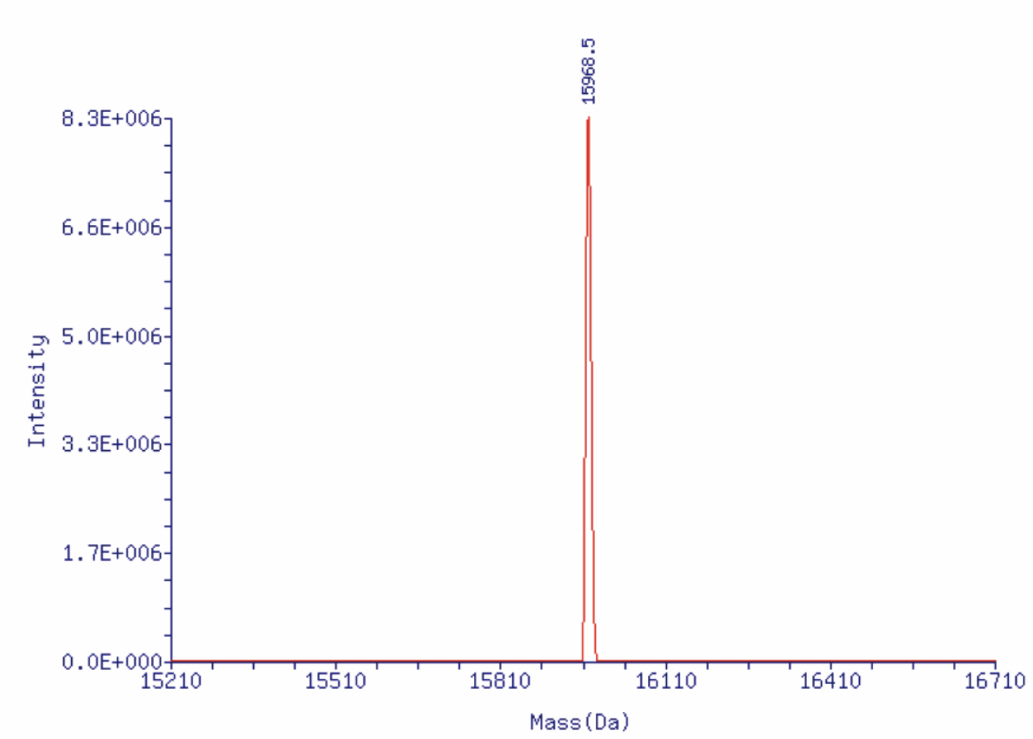


**Figure S15.** Mass spectrometry analysis of LH2-v2. Molecular weight is 15968.5 g/mol.


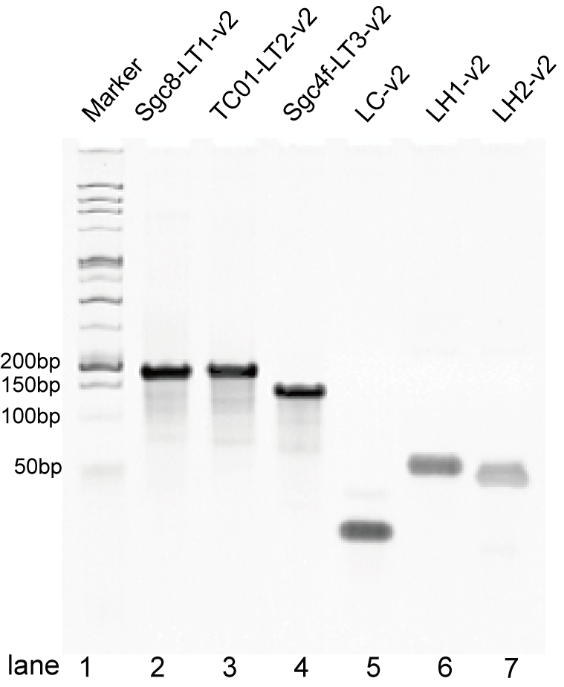


**Figure S16.** Urea PAGE analysis of L-DNA sequences of triple-aptamer-based logic device.


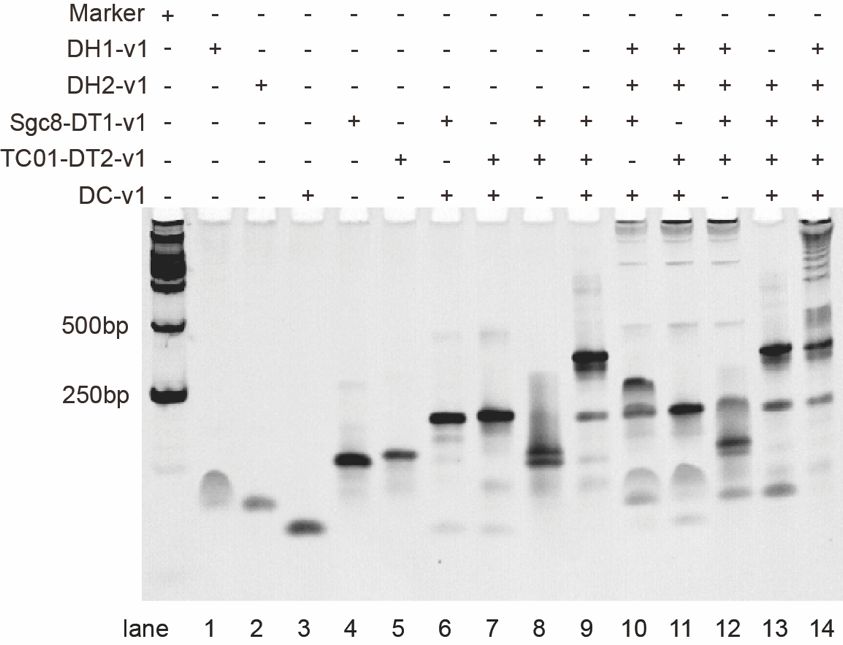


**Figure S17.** Native-PAGE analysis of the triple-aptamer-based associative activation of HCR with L-DNA sequences in solution.


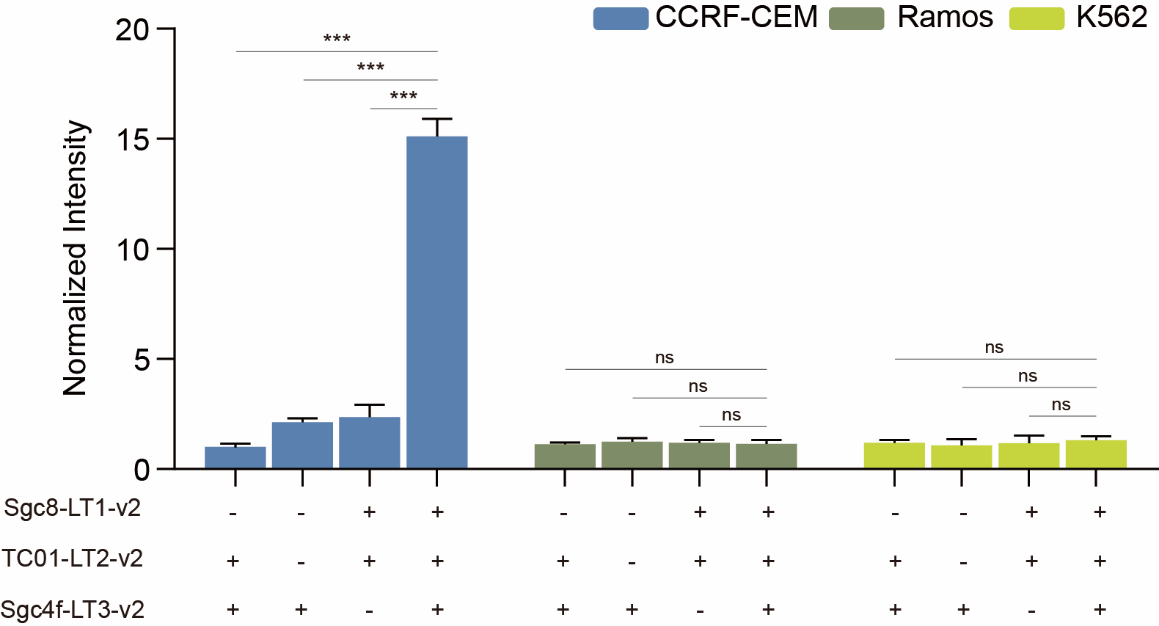


**Figure S18.** Statistical analysis of flow cytometry signals for indicated cell lines using a triple-aptamer-based logic device with L-DNA probes. Fluorescence intensity of cells incubated with H1 and H2 were used as background for normalization. P values were determined by two-sample equal variance two-tailed t-tests (***P < 0.001, **P < 0.01, *P < 0.05, ns = not significant).


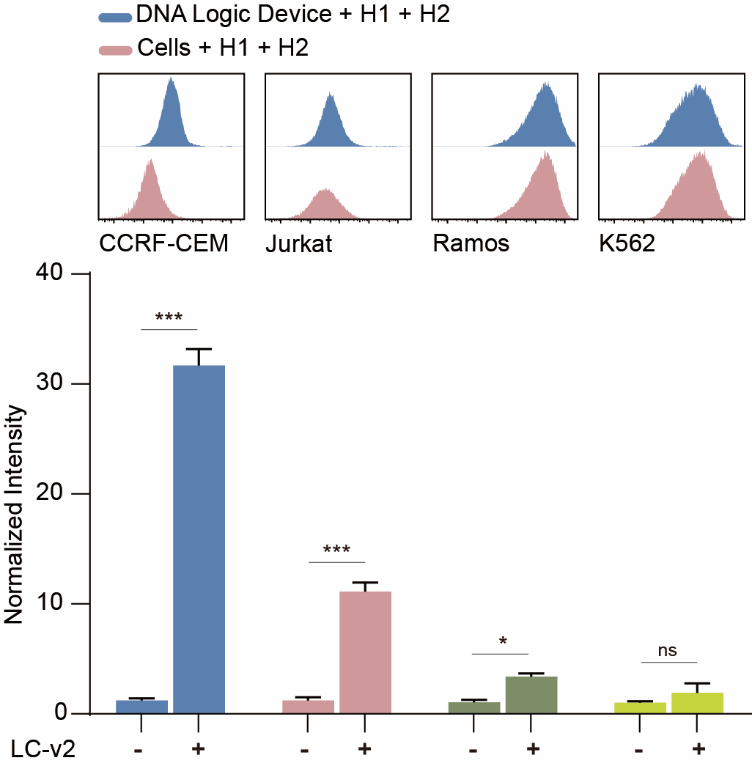


**Figure S19.** Representative and statistical analysis flow cytometry results of labeling signals on four different cell types using a triple-aptamer-based logic device with D-DNA probes. P values were determined by two-sample equal variance two-tailed t-tests (***P < 0.001, **P < 0.01, *P < 0.05, ns = not significant).


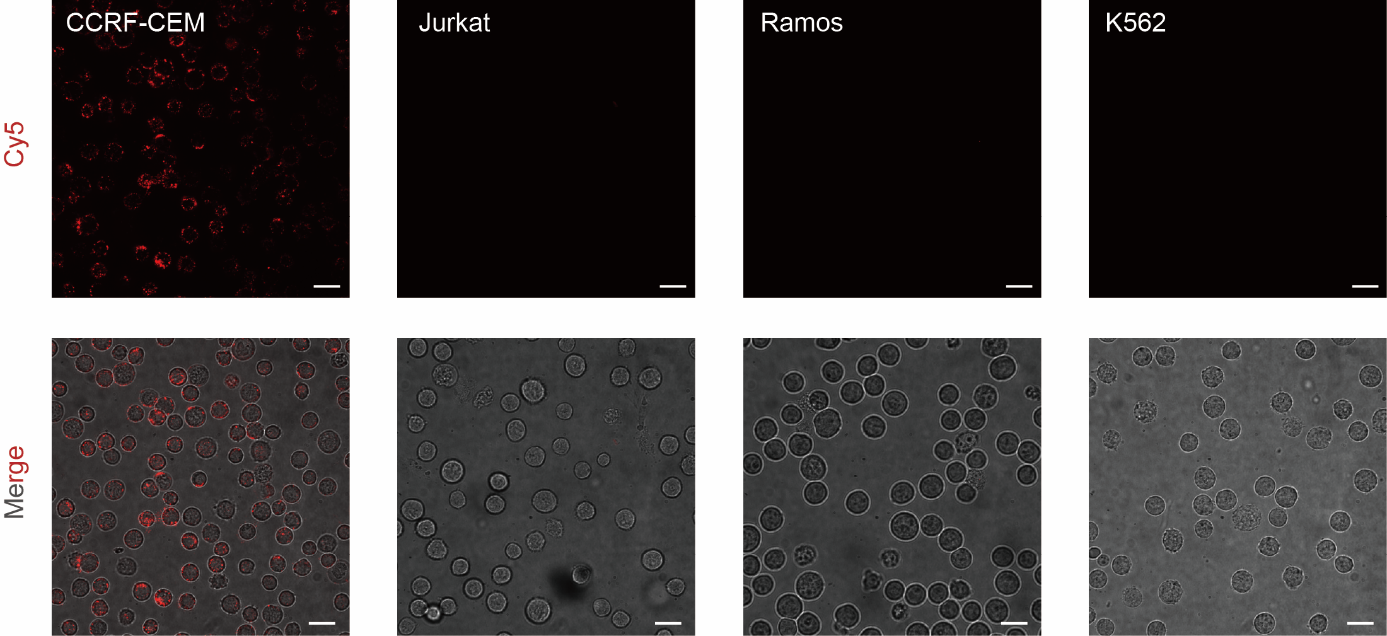


**Figure S20.** Confocal microscopy images of four different cell types with a triple-aptamer-based logic device using D-DNA probes. Scale bar indicates 10 μm.


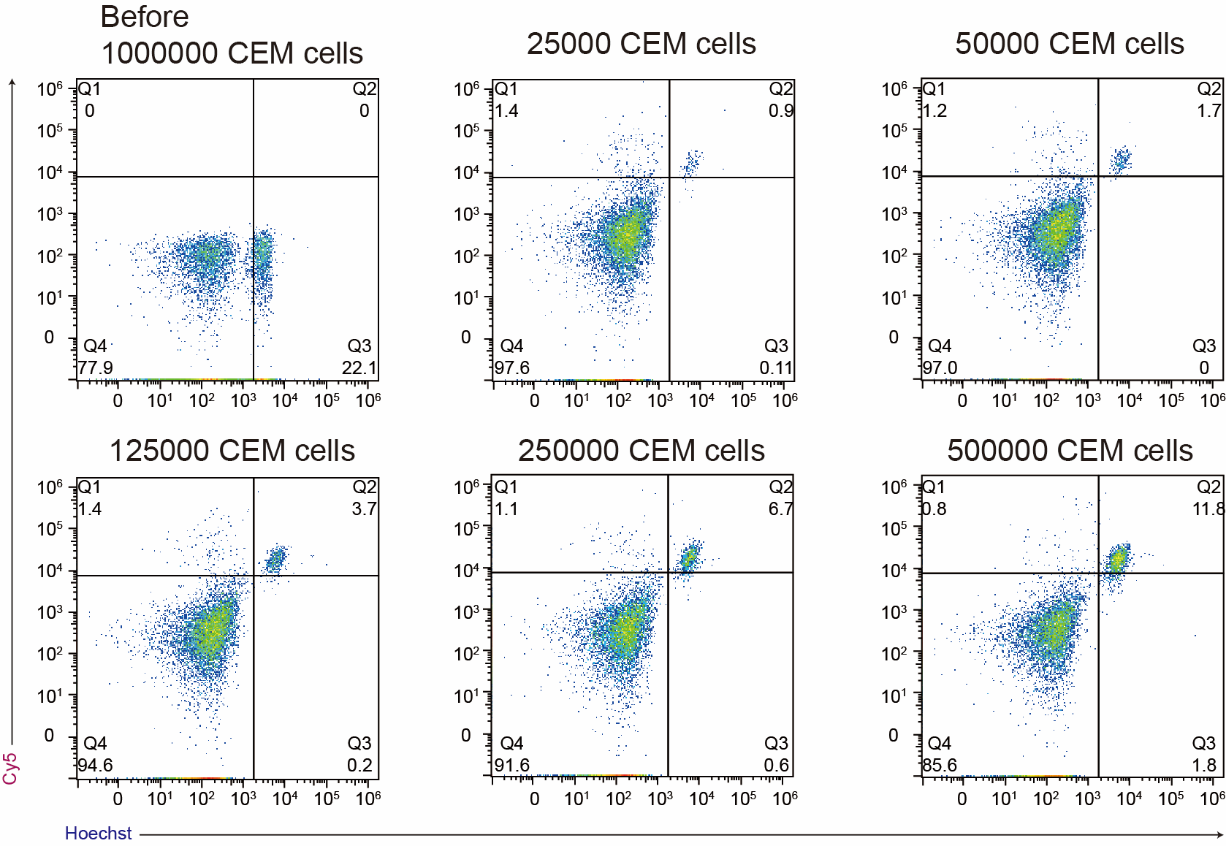


**Figure S21.** Identification of different concentrations of target CEM cells in real blood samples via a dual-aptamer-based L-DNA logic device. Different numbers of CEM cells (25000, 50000, 125000, 250000, and 500000) were prestained with Hoechst and added to 1 mL of blood.
